# Assimilating human feedback from autonomous vehicle interaction in reinforcement learning models

**DOI:** 10.1007/s10458-024-09659-4

**Published:** 2024-06-26

**Authors:** Richard Fox, Elliot A. Ludvig

**Affiliations:** University of Warwick, Coventry, CV4 7AL UK

**Keywords:** Preference learning, Autonomous driving, Human factors

## Abstract

A significant challenge for real-world automated vehicles (AVs) is their interaction with human pedestrians. This paper develops a methodology to directly elicit the AV behaviour pedestrians find suitable by collecting quantitative data that can be used to measure and improve an algorithm's performance. Starting with a Deep Q Network (DQN) trained on a simple Pygame/Python-based pedestrian crossing environment, the reward structure was adapted to allow adjustment by human feedback. Feedback was collected by eliciting behavioural judgements collected from people in a controlled environment. The reward was shaped by the inter-action vector, decomposed into feature aspects for relevant behaviours, thereby facilitating both implicit preference selection and explicit task discovery in tandem. Using computational RL and behavioural-science techniques, we harness a formal iterative feedback loop where the rewards were repeatedly adapted based on human behavioural judgments. Experiments were conducted with 124 participants that showed strong initial improvement in the judgement of AV behaviours with the adaptive reward structure. The results indicate that the primary avenue for enhancing vehicle behaviour lies in the predictability of its movements when introduced. More broadly, recognising AV behaviours that receive favourable human judgments can pave the way for enhanced performance.

## Introduction

A primary concern of automation is its ability to integrate with existing systems, which, when considering road usage, have a strong social element [1]. On top of optimising aspects of driving that naturally lend themselves to reward metrics, i.e., time to destination, fuel efficiency and lane discipline [2], vehicle automation must learn valid and useful behaviours when interacting with pedestrians and other road users [3, 4]. A key challenge researchers face in building such an agent is designing a suitable reward function to elicit such behaviours [5]. What are these behaviours, and how does one translate that to an RL algorithm? This paper addresses this question by developing a new methodology for embedding human judgements in an iterative-feedback loop for adjusting the reward function for an RL agent.

Most approaches to embedding human judgments into autonomous RL agents focus on the efficiency of an algorithm to minimise utilitarian measures, such as time to collision, time to destination and fuel efficiency [6, 7]. The system being analysed often has many complex inputs [8, 9], as an autonomous vehicle would have. Still, an equally complex social environment exists underneath [3, 4], which the agent can only access through the medium of human feedback. Having a noisy feedback signal makes the task of modelling complex social behaviour within the reward structure intractable, as the decision-making process of the people providing feedback is unknowable.

One approach to incorporating human feedback is through shaped reward functions. Reward functions are performance metrics, informing on the performance of a set of actions by typically summing the collected rewards. When a performance metric is not known, however, it is not clear what the reward for a set of actions should be [10]. The reward function must then be shaped—e.g., through terms that allocate reward based on selected combinations of environment attributes—to elicit the desired behaviours by rewarding the actions that constitute said behaviours. There are many potential pitfalls and inherent biases at play when performing reward shaping, such as naive design leading to unexpected behaviour, dependence on context from stakeholders in the environment, and inherently being a multi attributed problem, especially in the case of autonomous driving [5]. In autonomous vehicle optimisation, these can have many different forms built for several different paradigms [5]; our paradigm of interest is that of human preferences.

A preference-learning approach extends the concept of reward shaping to allow the agent to learn from an expert’s ranking over a set of actions, by shaping the reward to be weighted on those rankings. The basic approach is to learn a policy by eliciting feedback from people using a pair-wise trajectory preference, which serves as a measure of policy quality. This approach, however, often requires experts in narrow and highly skilled domains. To alleviate the limitation of pair-wise preferences to inform on policy quality, there has been work that allows for the agent to use the expert’s rankings to extrapolate new behaviours [11]. By seeking explanatory patterns for the preferences, the best policy even exceeded the best performance that was demonstrated. In other work, Jain et al. [12] allowed their model to learn context-dependent behaviours much more efficiently, using a reward-based utility function without an explicit cost function to be optimised. Instead, their model used the preference feedback to compute a gradient of the utility parameters directly, permitting the policy to be defined by the RL agent. Creating such a utility function can be especially useful for the current project because feedback from pedestrians is not always consistent, deterministic, nor even stationary.

When directly considering how pedestrians interact with AVs, Suresh et al. showed that pedestrians’ acceptance of AVs depends on their trust in the AVs [13]. They conducted an objective-based evaluation of behaviours [14], in terms of safety, performance, and comfort, with 30 human participants in a virtual-reality environment. They found that pedestrians’ trust in AVs was influenced by AV driving behaviour as well as the presence of a signal light. In [14], the authors propose a model representing the three objectives—safety, efficiency, and comfort—by the weights of a linear regression of observable variables, including latent trust variables and other experimental considerations. The intuition behind these objectives is that driving behaviours can be characterised by weighting values, especially as there is a competing optimal weighting between AV passengers’ preference and the pedestrian’s preference, both of which should be optimised.

Our approach combines both a preference-learning structure and a behavioural-evaluation structure. The approach starts with the simplest reward to produce driving behaviour and uses the empirical evidence from safety judgments by humans to inform reward shaping. As a result, the approach works towards the goal of autonomous behaviour that makes pedestrians feel safe. Improving the behaviour by highlighting the behavioural aspects missing in the process, with a view to be included in future work to improve the overall rating of the behaviour by pedestrians.

The present research develops a methodology to directly ask what behaviour pedestrians find suitable by collecting quantitative data that can be used to measure an algorithm's performance and helps better align the performance of an AV with its users’ preferences. The paper then shows improvements, in the judgement of the pedestrians, in “human-like” behaviour when such systematic feedback is collected from human agents and embedded into the reward function. The technique harnesses a formal iterative feedback loop using computational RL and behavioural science techniques, where the reward structure is adapted by eliciting behavioural judgements collected from people in a controlled environment. The policy trained with the updated reward is then shown, without explicit participant knowledge, back to the participant to assess the updated behaviour. The reward function is based on three terms gleaned from pilot experiment data [6], in which participants were asked to judge aspects of the vehicle’s behaviour. Based on the current human feedback, an agent that was trained with each one of these three terms in the reward function altered to match that feedback is selected, and the behaviour of the resultant policy is then presented for judgement in another round of feedback.

Agents were trained with Deep Q Network (DQN) [15] models as we wish to use the simplest methods to control complications around inference of what is altered by changing the reward function and on what participants are giving judgements. Given that the state space of our custom Pygame [16] simulator^1^ is large, based on pixel coordinates, we still require a deep approach as the simpler tabular methods would be computationally intractable. The DQN was trained on a simple Python-based Pygame pedestrian crossing environment, and the reward structure was adapted by eliciting behavioural judgements collected from people in a controlled environment. We elicit participant judgements, which are subjective ratings on vehicle driving quality, rather than eliciting a preference between multiple given vehicle trajectories. Participants, thus, are not considered experts; we regard them as normal pedestrians and guide the reward function by their judgements.

## Methods

### Simulator design

In developing and testing autonomous vehicles, simulators play an essential role in providing controlled environments for experimentation. To address the specific research requirements, a custom driving simulator was developed using Pygame, a cross-platform set of Python modules designed for writing video games. Leveraging the capabilities of the Pygame library was instrumental in defining the basic entities within our simulation environment, which operates on Pygame’s grid system at its core. This simulated driving environment was structured to conform to OpenAI Gym's specifications to enhance the simulator's compatibility with a range of reinforcement-learning algorithms. The custom simulator offers a blend of simplicity and functionality meeting the specific needs of our research.

**Fig. 1:**
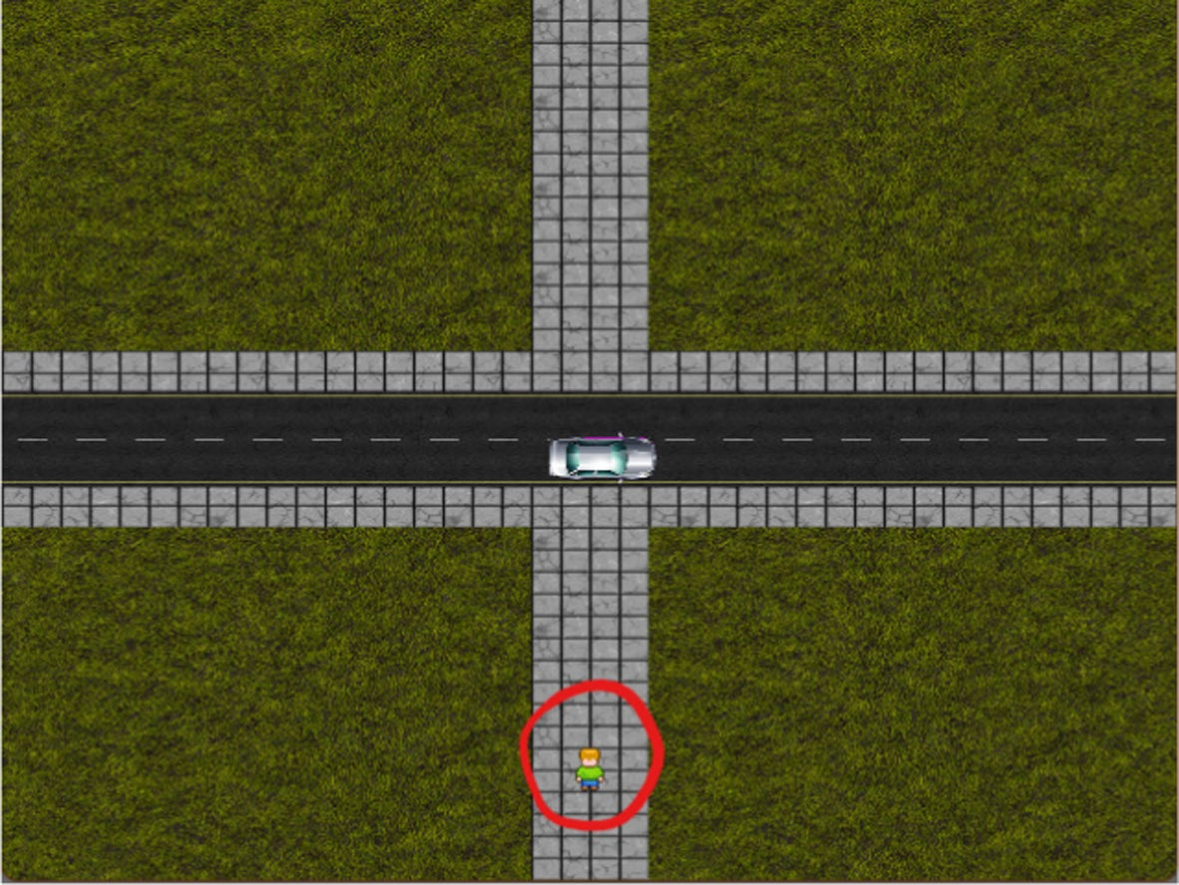
Rendering of custom Pygame driving environment using open-source assets. Circled in red is the pedestrian sprite that is controlled by experiment participants

The simulator was designed with two primary modes of operation: a render mode and a headless mode. Figure 1 depicts the rendered display as shown to participants in behavioural experiments. The render mode was developed for visualising the interactions between the autonomous vehicle and the pedestrian. This visual representation provides a user-friendly interface where participants can visually discern the road, the car, and their avatar as a pedestrian. Open-source assets^2^ were employed in this mode to ensure an aesthetically pleasing environment, facilitating participant immersion in the simulation. Conversely, the headless mode operates without any graphical display, offering a significant advantage during the training phase of the autonomous vehicle. In this mode, the environment was essentially reduced to four primary rectangles representing the pedestrian, the car, and the vertical and horizontal stretches of the road. The vehicle's observations within this environment were straightforward yet effective. The vehicle perceived bounding rectangles, or ‘Rects’, for both itself and the pedestrian. These Rects were defined within Pygame's internal grid system, ensuring precision and consistency in the vehicle's perception. Additionally, the vehicle was provided with a clear goal, indicating the coordinates it should aim for from its starting position. The simulated actions were defined as one of the four cardinal and four intercardinal directions at a speed of 1, 2 or 3 grid-squares/pixels per computational time step or a no-move action resulting in 25 total actions.

The other important modelling features, in the context of the simulator, were if a vehicle collides with a pedestrian, the episode concludes immediately and a large negative reward $\left(-\infty \right)$ is allocated to that state. In addition, agents were granted an augmented positive reward that made their total reward exactly 0 when they intersected with their designated goal coordinates and negative rewards otherwise. The DQN loss function was computed over actions with a lower cost for actions considered best. As the action space is discrete with negative rewards, there was the possibility of an infinite valued loss-term arising in the event of a pedestrian collision. The model avoided such infinite-valued loss-term issues because there was always an action that avoided a collision by having pedestrians limited to a move speed of 2 while the vehicle could move at a speed of 3. Combined with either the shaped or constant reward, there was always an action better than colliding with a pedestrian to which the policy could converge. When the task was successfully accomplished, the reward given was positive and matched the absolute value of the smallest possible reward the agent could obtain while still reaching the goal, meaning that a spatially optimal trajectory received a reward of 0.

Additionally, the simulator considered only two sets of starting points and goals, representing driving directions from right to left and vice versa. The maximum episode duration was set to the distance to the goal plus one time step. This constraint ensured that the agent-controlled vehicle can reach its destination by moving at the slowest pace in a direct path. By implementing a negative reward system and concluding the task upon successful completion, this approach ensured that agents were motivated to identify the most efficient routes. This approach aims to terminate episodes swiftly, thereby reducing the accrual of negative rewards.

During the preliminary stages of model training and testing, the DQN models struggled to develop an effective driving policy. The models exhibited a tendency to achieve only slight improvement in rewards without demonstrating any genuine driving behaviour. A common tactic adopted by the models was to drive off-screen, thereby avoiding the accumulation of penalties immediately. Rather than further refining the reward structure to address this challenge, which could potentially obfuscate the desired behavioural outcomes, a technique known as action-masking was implemented [17]. Action-masking restricts an agent’s choices by allowing it to execute only valid actions in a given state. To facilitate this constraint, the simulator was enhanced with built-in action masking functionality. This addition ensured that for every state presented to the agent, a corresponding action mask was provided. Actions that would result in the vehicle exiting the screen were deemed invalid and were consequently masked out, preventing the agent from selecting them. This approach aimed to guide the DQN models towards more realistic and effective driving behaviours without overly complicating the reward structure.

### Reward design and RL training

The agent’s goal is to find the best set of actions $A$ given the states $S$ visited, referred to as the optimal policy ${\pi }^{*}\left(s\right),$ where policy function $\pi$ returns an action $a\in A$ to be taken in the state $s\in S$ of the environment of interest. A trajectory ${\tau }^{\pi }=\left\{\left({s}_{t},{a}_{t}=\pi (s\right)\right\} \forall t\in \left\{\text{1,2},\cdots ,T\right\}$ is the set of state-action pairs returned by policy $\pi$ for a given terminated instance of environment interaction over time $T$. The total expected return from a trajectory is:

1 $$R_{{\tau^{\pi } }} = \mathop \sum \limits_{t = 0}^{T} r\left( {s_{t} } \right)\quad \forall \left( {s_{t} ,a_{t} } \right) \in \tau^{\pi } ,$$

which is general to the theory of RL. For this work, however, we also consider a shaped reward, based on the inter-action vector, decomposed into reward for relevant behaviours. The reward function is determined by three terms, providing rewards for speed ${r}_{\nu }$, proximity to destination ${r}_{\delta }$, and rate of heading change ${r}_{\Delta }$. These are summed and scaled to give us the full reward for a given state $r\left(s\right)$:

2 $$r\left( {s_{t} } \right) = r_{\nu } + r_{\delta } + r_{\Delta } - 3 = \frac{{\nu_{t} }}{{\nu_{max} }} + \left( {1 - \frac{{\delta_{t} }}{{\delta_{max} }}} \right) + \left( {1 - \frac{{\Delta_{t} }}{2\pi }} \right) - 3,$$

where ${\nu }_{t}$ is the speed of the vehicle at time $t$, and ${\Delta }_{t}$ is the amount of heading change or the amount of steering at time $t$. Both terms are determined by the previous action ${a}_{t-1}$, reflecting a dependence on the previous state-action pair as Markovian dynamics allows. The term ${\delta }_{t}$ is the distance from the state that the vehicle agent is currently in (${s}_{t}$) to the agents given destination. Each of the three terms are in the interval $(\text{0,1}]$ with $1$ being the optimal value, and we apply a negative $3$ to our reward to ensure that the reward is always negative, making an expected reward of $0$ the theoretically optimal solution.

All three of these quantities are required to be within the range $\left[\text{0,1}\right]$, to avoid violating constraints for optimisation that we discuss further below, and we restrict this further to $(\text{0,1}]$ to avoid numerical errors in computation with variables that are exactly $0$. As such, they are normalised by their maximums, ${\nu }_{max},{\delta }_{max}$ and $2\pi$ in order of appearance in Eq. (2). The term ${\Delta }_{t}$ is a radian angle, so ${\Delta }_{t}\in \left[\text{0,2}\pi \right)$. The other 2 terms are set by environment variables, which are effectively hyperparameters that could be explored. For the purposes of this study, however, they are set to fixed values as the best environment hyperparameters are not of particular interest. Also, to accurately reflect the task, close distances from the goal and smooth/slight steering should be rewarded more, so both ${\delta }_{t}$ and ${\Delta }_{t}$ are negated, as the task requires minimising these quantities whilst maximising reward.

The reward structure, as described in Eq. (2), was designed to capture three critical aspects of the vehicle's behaviour: its speed, its proximity to the goal, and the smoothness of its steering. Each of these aspects plays a distinct role in determining the overall performance and safety of the vehicle. In essence, while distance serves as a foundational metric, speed and steering encapsulate both basic and advanced driving behaviours. The dual nature of the speed term, where it can belong to either category, highlights its versatility in capturing different facets of driving. The reward structure, therefore, provides a comprehensive evaluation of the vehicle's performance, balancing both essential and subtle driving behaviours.

**Fig. 2:**
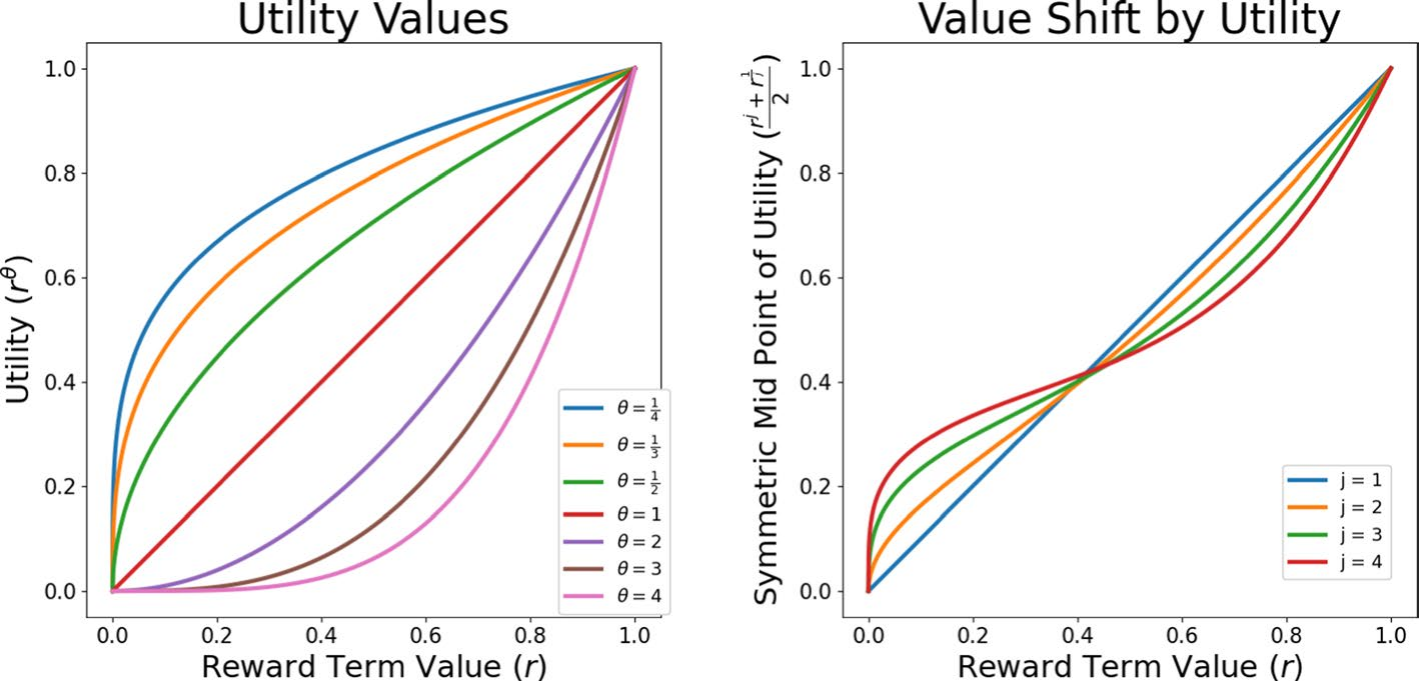
The left panel shows the effect of applying the utility to a single term $r$, where low values of exponent ${\theta }_{i}$ for any given term dampen high rewards and high values of the exponent do the converse. The right panel shows the difference between inversely valued function exponents compared to the linear case. The graph shows how the dampening effect of a concave utility is greater on rewards above $0.5$ than the convex counterpart, thus undermining the current policy and encouraging adaptation. The converse is also true, indicating that for rewards below$0.5$, the increase from the convex utility function outweighs concave dampening, thus reinforcing the current behaviour in all circumstances

Our approach uses utility functions (see Fig. 2) as an alternative to traditional reward models. These utility functions are transforms of the reward function, which directly influence the system. Instead of altering the inherent task reward, we adjust the reward to align with individual preferences. Utility takes up a role of direct influence in the place of a reward model but is not a reward model; the utility shifts the reward function to match the individual, but does not change what the task reward is. By leveraging utility, we aim to discern implicit preferences derived from judgments, thereby facilitating both implicit preference selection and explicit task discovery in tandem.

We apply a utility transform ${U}_{\theta }$ to each term, which takes the form of a parameterised exponent:

3 $$\begin{aligned} U_{\theta } \left( {r\left( {s_{t} } \right)} \right) & = r_{\nu }^{{\theta_{\nu } }} + r_{\delta }^{{\theta_{\delta } }} + r_{\Delta }^{{\theta_{\Delta } }} - 3 = \left( {\frac{{\nu_{t} }}{{\nu_{max} }}} \right)^{{\theta_{\nu } }} - 1 + \left( {1 - \frac{{\delta_{t} }}{{\delta_{max} }}} \right)^{{\theta_{\delta } }} \\ & \quad - 1 + \left( {1 - \frac{{\Delta_{t} }}{2\pi }} \right)^{{\theta_{\Delta } }} - 1. \\ \end{aligned}$$

The advantage of parameterising each term is that it enables feedback to be elicited for each term individually, allowing each term to be non-linear over a trajectory, but decompose into a linear sum at a policy execution level. As we do not want to yield a complex valued reward, the negative domain shifting is also decomposed and applied to each term individually, importantly after applying the parametrised exponent.

Figure 2 illustrates how these utility parameters transform the reward space and informs as to why the construction of the reward requires such care to remain within the range $\left[\text{0,1}\right]$, ensuring a symmetric and bounded variation. At policy execution, we can still return a sum of 3 scalar terms only depending on the previous state, therefore not violating either of the assumptions of Markovian and sub-game optimal dynamics for learning. Hence the nonlinearity is applied to expressions in the bounded positive interval $(\text{0,1}]$ (Fig. 2), before other scaling is applied. As a result, the utility function mathematically cannot violate this assumption as it remains within this bound, respecting the linear optimality by preserving monotonicity and only altering the asymptotic nature of the function. This utility function therefore acts as a filter over the reward and can be optimised directly but statically, only learning a policy for one fixed set of parameters. When the utility is higher than the base linear reward, rewards increase across all states with the effect compounding as the linear reward increases, effectively reinforcing the current policy’s behaviours. The converse is true for low ratings, which encourage exploration of different behaviours as the policy reconverges. The linear form of the utility, equivalent to the standard shaped reward, is used as a pre-trained model from which to train each utility variant model for a further fixed number of episodes.

For the training of agents, the framework of choice was Stable Baselines3 (SB3) [18],^3^ a popular library that offers a variety of reinforcement-learning algorithms. A notable limitation of SB3, however, is its lack of support for action-masked DQN, a crucial feature for our simulator. Thus, adaptations to the standard SB3 DQN implementation were needed.^4^ We used a simulation grid with dimensions of 640 × 480 pixels. The training process was initiated with two distinct DQN models. The primary model was trained using our specially designed reward-shaping mechanism, while another model was trained with a consistent reward for each time step. When the vehicle deviated from the road in the simulation, the fixed reward was marginally decreased. The magnitude of these fixed rewards was deliberately chosen to align with the rewards from our shaped function, facilitating a more straightforward comparison between the two models. The primary purpose of the baseline shaped model, where all parameters were set to zero, was to serve as a foundational model. From this baseline, variant models were trained, each for a predetermined number of episodes. These variant models were derived by multiplying the utility-parameterised three-term reward function with a five-point Likert scale, resulting in a range of 125 parameter sets for the experiments.

### Behavioural experiment design

The feedback-collection experiment was conducted in a controlled environment within the behavioural science lab in the Psychology Department at the University of Warwick. There was a total of 124 participants in the experiment. Experiments took up to 30 min, and participants received course credit for participating. All participants were current University of Warwick students who received no prior information on the experimental task beforehand. No information on protected characteristics of the participants was collected. All participants provided informed consent, and the research was approved by the Department of Psychology Research Ethics committee. All anonymized data are publicly available at: https://osf.io/mgser/.

**Fig. 3:**
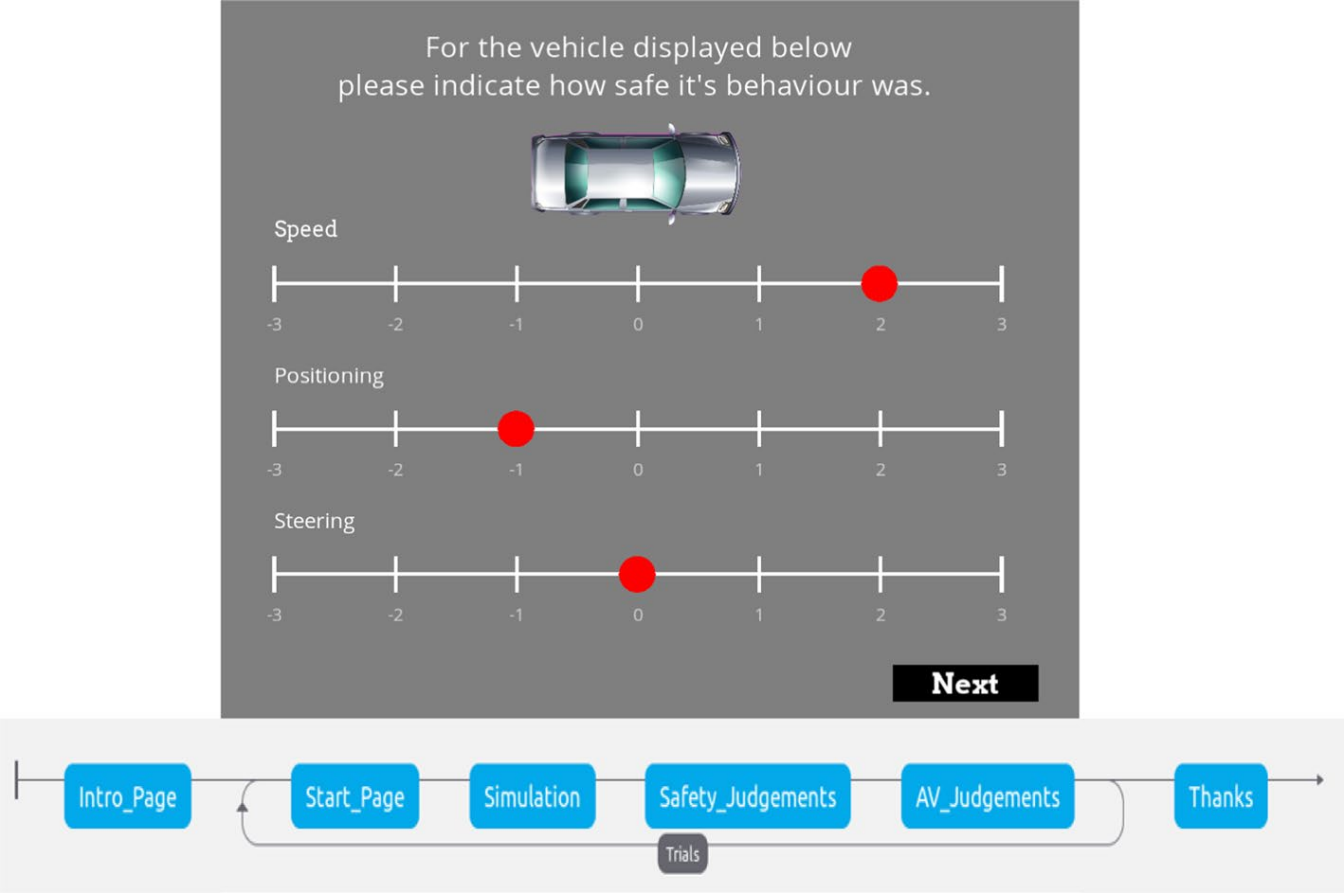
Safety Judgement stage of the behavioural experiment procedure with scales for each reward aspect. Along the bottom is a schematic of the entire experimental process. From left to right, the task started with an explanation of the task shown only once, then a simulation where the interaction happens, followed by Likert-scale responses which form the trials that are repeated for the duration of data collection

Participants were invited to the lab, with 24 PCs all individually partitioned, where they were seated in front of a PC and presented with an instruction sheet. Each session accommodated up to 12 participants seated at least 1 PC apart with instructions not to communicate during the experiment. Upon arrival, participants were given a brief overview of the study's objectives and what was expected of them during the session; they also were asked to sign for their consent for their data to be collected, anonymised, and shared before commencing. Following this introduction, the trained models were presented in the Pygame simulator (see Fig. 1). Participants provided responses judging the safety of different aspects of the AV behaviour (see Fig. 3). These responses were obtained with three different initialization seeds, ensuring variability in the scenarios and interactions presented to the participants. Aggregating the feedback, each participant contributed a total of 200 feedback data points, consisting of 4 points per trial over 50 trials each displaying a scenario for between 10 and 25 s. Of these 200 data points, 150 were direct evaluations related to the utility parameters, while the remaining 50 were judgments about the likelihood of the agent being an AV.

Each participant went through a structured procedure comprising five blocks of trials, with each block containing 10 individual trials before a reset to the linear model configuration for the next block. During each trial, participants were prompted to provide feedback through the three safety ratings. Additionally, after every trial, participants were asked to make a judgment about whether they believed the agent's behaviour was more characteristic of an AV or a human driver. To elicit feedback from human participants, an interactive experiment^5^ was set up using PsychoPy [19]. In this experiment, participants took on the role of a pedestrian within a simulated crossing environment, as depicted in Fig. 1. Their task was to navigate this environment while interacting with the various models. Integrating the Pygame environment into a PsychoPy experiment enabled participants to experience the agent trained with a given reward function. Participants then provided safety ratings on each of the 3 aspects considered in the reward design in a seamless loop. This setup allowed the parameters for subsequent models to be selected based on feedback from the previous trial. The only verbal anchors to the scale were safe/unsafe at the positive and negative extremes of the scale respectively. This safety judgements are not necessarily comparable between participants, as each participant could have a seemingly neutral behaviour in differing positions on the scale. Instead, the judgements provide a relative baseline that we look to improve for each participant leading to improvement being assessed on aggregate. Even so, the feedback was very noisy, especially in terms of participant intent, and it is therefore more appropriate to treat the feedback as a stochastic process where analysis of single instances lack descriptive power.

To ensure consistency and estimate uncertainty, participants were reintroduced to the linear model every 10 trials, restarting the process of selecting subsequent parameters through safety ratings. This approach created a consistent baseline for comparison, providing a consistent reference point for participants throughout the experiment and aided in pinpointing any potentially insincere feedback. The 7th trial in every set of 10 trials featured a random, non-shaped reward trajectory. These trajectories were either from researcher-controlled vehicles or derived from the fixed-reward model. Additionally, after each trial, participants were asked to judge whether the vehicle was human-controlled or operated by an AV. By collecting judgments on human-like behaviour, we aimed to discern if trajectories deemed “expert” were indeed superior based on participant evaluations. To further refine our study and account for another potential bias, we introduced variability in vehicle colours. With a palette of eight colours, the vehicle's hue was randomly selected each time the simulator was run, and this colour data was stored alongside other trial information. This measure was implemented to identify and account for any colour-based biases in judgments, such as the perception that red cars, often associated with sportiness, might be inherently riskier.

Feedback was gathered from the human participants by asking them to evaluate specific interactive trajectories. Their judgments were recorded on a 7-point Likert scale ranging from − 3 (very unsafe) to + 3 (very safe). A 5-point scale would not have had enough intervals to include an interval for 0 as the linear utility parameter, so to ensure the scale remained symmetric around 0, a 7-point scale was used. After gathering this feedback, participants were then presented with a new interactive trajectory. This trajectory was produced by a utility-adjusted reward function that matched their initial judgment, as described in Eq. (3). This relationship allowed a mapping from the integer Likert-scale points (LP) to the parameterised exponents in Eq. (3) directly, according to:

4 $$\theta_{i} = \left\{ {\begin{array}{*{20}l} {sgn\left( {LP_{i} } \right) \cdot 2} \hfill & {\quad \left| {LP_{i} } \right| \ge 1.8} \hfill \\ {sgn\left( {LP_{i} } \right) \cdot 1} \hfill & {\quad \left| {LP_{i} } \right| \ge 0.6} \hfill \\ 0 \hfill & {\quad Otherwise} \hfill \\ \end{array} } \right..$$

At ${\theta }_{i}=0$, this equation reverts to the non-parameterized reward function, as shown in Eq. (2). The structure of Eq. (4) ensures that higher ratings correspond to increased utility, especially when the utility mirrors the form of ${U}_{\theta }$. When we consider a three-term reward/utility function combined with the reduced seven-point Likert scale (see below), we obtain $125$ (or ${5}^{3}$) distinct parameter sets. These five sets are derived from the Likert scale's range of $-3$ to $3$, which was divided into five distinct intervals: $[-3,-1.8]$, $(-1.8,-0.6]$, $(-\text{0.6,0.6})$, $[\text{0.6,1.8})$, and $[\text{1.8,3}].$ Each of these intervals corresponds to an integer parameter ranging from $-2$ to $2$ in consecutive order. The resulting function retains its symmetry and exhibits convexity for positive LP values and concavity for negative LP values, as depicted in Fig. 2. This approach ensures a systematic and mathematically sound method of correlating human judgments with specific utility parameters.

Algorithm 1 specifies how, during the behavioural experiments, participants were exposed to policies that reflect adjustments in parameters, guided by their LP judgments. The participant thus effectively picked the subsequent model from the pre-trained set with which they then interact. By analysing these selection data, we aimed to identify the utility that aligned most closely with their perception of a superior-performing agent. While individual preferences naturally vary, often significantly, any consistent trends observed in aggregate data can validate our parameter choices. We pre-emptively trained models for all potential parameter sets. This enabled us to present participants with agents that appeared to adjust in real-time based on their feedback, without the need for extensive re-training in real time. Participants' judgments were not based on passive observation of a scenario. Instead, the participant actively controlled the pedestrian in the environment, and so they based their evaluations on direct interactions with the agent rather than just observing a predetermined trajectory. This active engagement can lead to unique instances, revealing a broader spectrum of the policies' learned behaviours, even when utility parameters are similar or identical.

**Algorithm 1:**
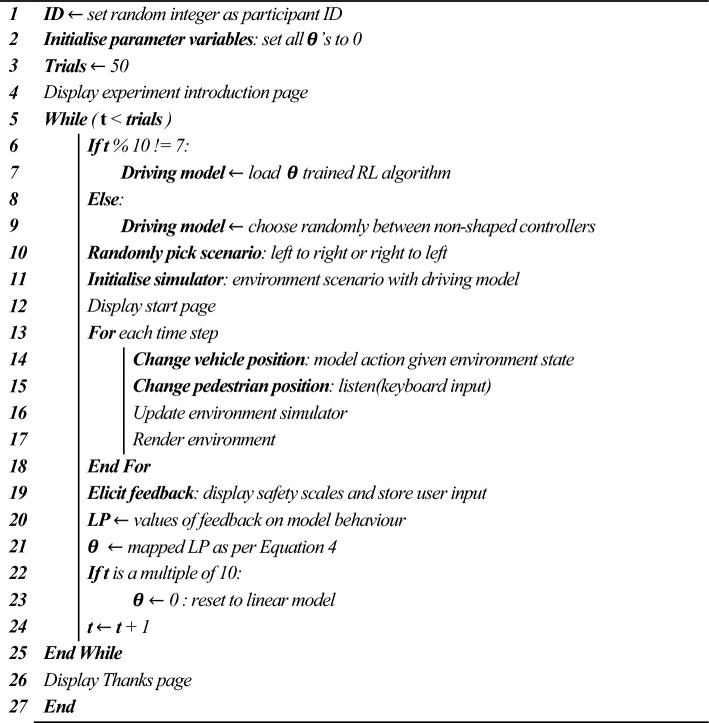
Trial-to-trial utility parameter updates

## Results and discussion

### Reward selection

**Fig. 4:**
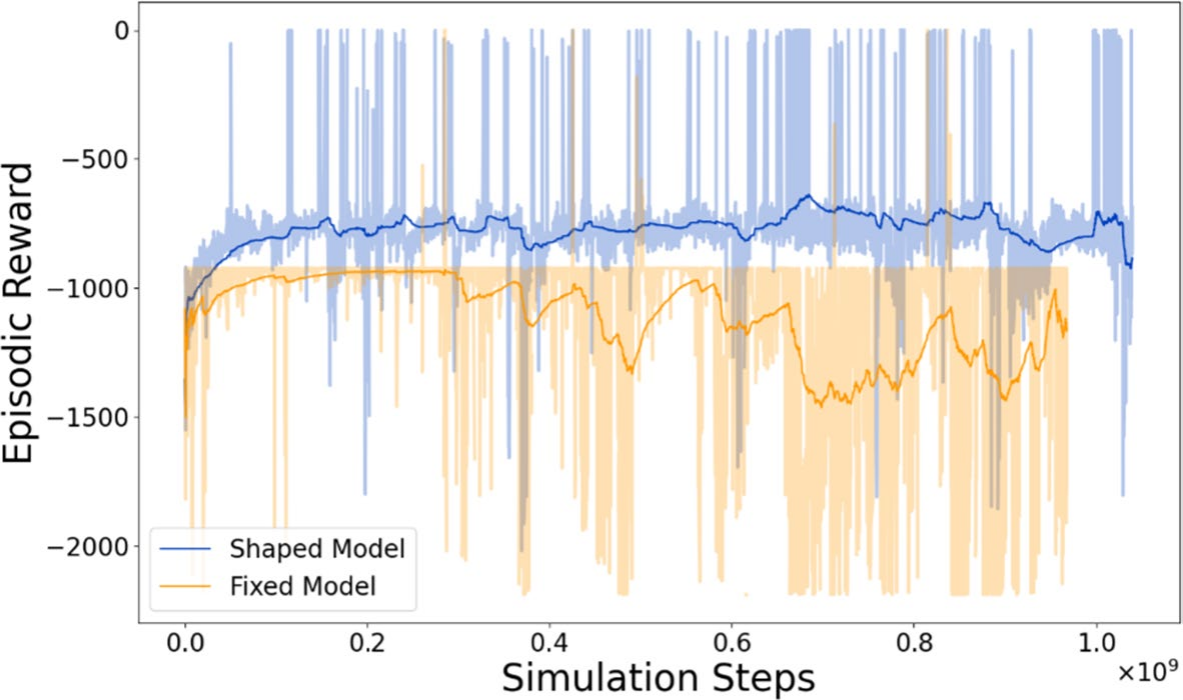
Reward curves for both the shaped and fixed reward models. Both were trained for 1.5 M episodes. An episode was at most 641 simulation time steps; here we plot the cumulative time steps for all episodes during training. Reaching the destination or colliding with the pedestrian ended the episode early, so while models completed the same number of episodes, the total number of timesteps differs. The solid line is the running average over 200 episodes

The first objective an agent must learn is the path to the goal, and Fig. 4 shows that this takes a much longer time when the reward is not shaped to guide the agent's progress in the sparse reward regime. To learn that heading to the goal improves the return, the agent had to avoid a pedestrian with noisy movement that was heading perpendicular to them. The shaped reward, Eq. (3), helps with this learning by giving a reward based on behaviours exhibited rather than just reducing reward by a fixed value at each time step. However, a straight-line path still had a high probability to intersect with the pedestrian in both the shaped and fixed reward cases. Therefore, there was always a trade-off between an efficient route with high reward and the possibility of hitting the pedestrian. Honing in on early successes too strongly possibly led to failures and hopefully caused the shaped agent to change its policy to find safer behaviours.

The optimal policy at this stage was simply reaching the destination as fast as possible while avoiding the pedestrian; however, when trying to optimise with human feedback it was not possible to define further aspects of the optimal policy. Without explicit, a priori knowledge of what made each individual participant feel that a particular behaviour was safe, it was impossible to better define what a policy (or reward function) optimised for pedestrians perception of safety ought to have been. Furthermore, participants may not have been able to directly report such information with any accuracy, nor consistency. Both this lack of a priori information and uncertainty over human feedback information motivated an extension of RL training with direct interaction with human participants to better incorporate agent behaviours that were perceived as safe by road users, beyond the improvements in efficiency observed with the reward shaping.

### Behavioural experiment

**Fig. 5:**
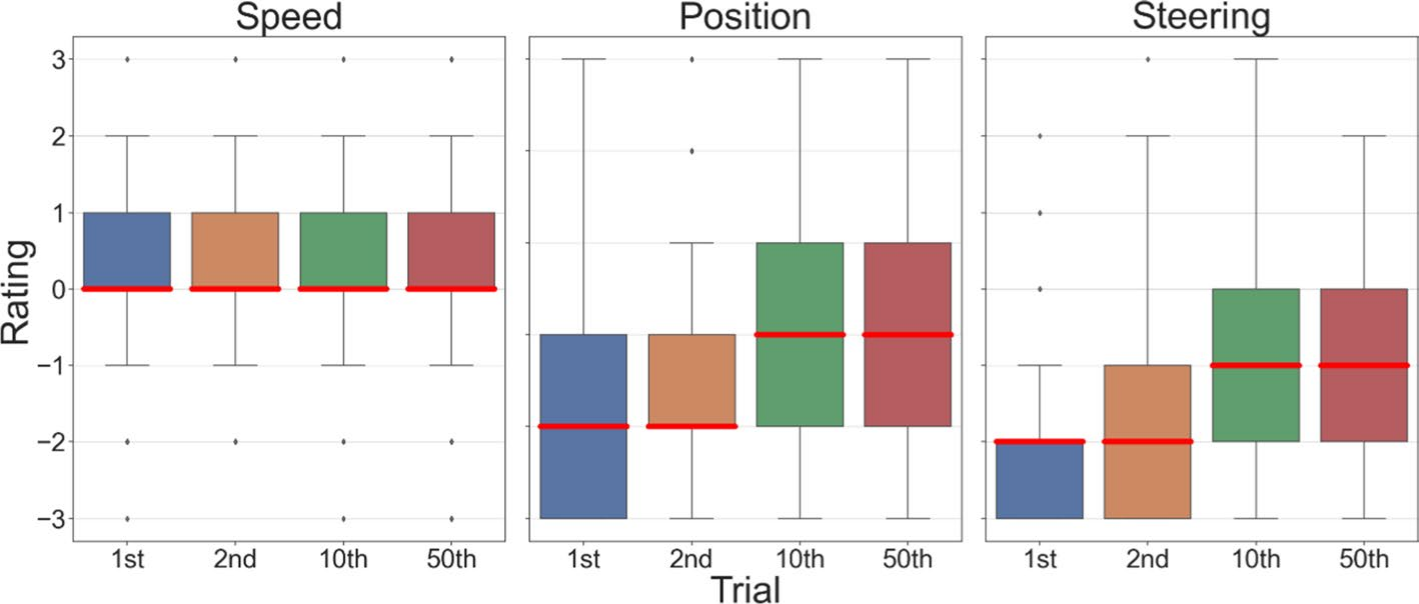
Participants’ aggregate ratings for the first two trials, the 10th, and the last trial, grouped into the feedback elicited for each term in the reward structure. The bold red lines indicate the median of the aspect rating for the respective trial. Because the values were discrete, the median and a quartile often have the same value. There was no effect in the speed term, but large initial improvement in the other two terms. There were, however, no further improvements beyond that initial bump

Figure 5 shows how, for some measures, there was a positive trajectory through the initial round, suggesting that the overall system was adapting to the feedback and selecting AVs with behaviours that were more in line with participant preferences. The speed of the agent was judged to be moderately appropriate from the outset, and this assessment remained consistent throughout the trials $\left(Mdn=0, {\upsigma }_{n-1}=1.14\right)$. These judgments suggests that the initial model's speed parameterization was already in line with participants' expectations. In contrast, the other two behavioural aspects started with relatively poor ratings for both position $\left(Mdn=-1, {\upsigma }_{n-1}=1.3\right)$ and steering $\left(Mdn=-1, {\upsigma }_{n-1}=1.27\right)$. Notably, there was a discernible improvement in the ratings for position and steering right after the very first interaction. Over the course of the first 10 rounds, these ratings improved more strongly on average, transitioning from poor $\left(Mdn=-1.33, {\sigma }_{n-1}=0.77\right)$ to moderate $\left(Mdn=-0.66, {\upsigma }_{n-1}=0.92\right)$, with this improvement being statistically significant under a two-sided Z test with a null hypothesis of equality, $Z(123)=-2.85,p=4.65\times {10}^{-3}$. This result indicates that where there was room for improvement, and the system based on the utility model was indeed responsive to feedback and made significant strides in these areas.

The utility model thus demonstrated enhancement when matching utility parameters with judgments, particularly during the initial trials. Interestingly, there is the possibility that as the model tried to diverge from this state in search of further improvement, it might have been perceived negatively, leading to poorer ratings. By incorporating the utility into this paradigm, each piece of feedback used contributes to finding the best behaviour without prescribing what that behaviour should look like. This approach selects for higher performing behaviours that assimilates better into the social climate. Therefore, judgements are not an alternative reward signal, but the reward signal that fully represents the underlying problem and is the task we want to achieve success in. While it is theoretically possible to embed any judgements using this approach, non-passive judgements are recommended, where the participants are in control of an entity in the environment and judging based on their interactions with the agent, instead of simply observing trajectory (as in [19]). Participant control leads to each instance being unique and showing a broader range of the policies’ learned behaviours, even with the same or similar utility parameters, than the observation of a fixed representative trajectory.

**Fig. 6:**
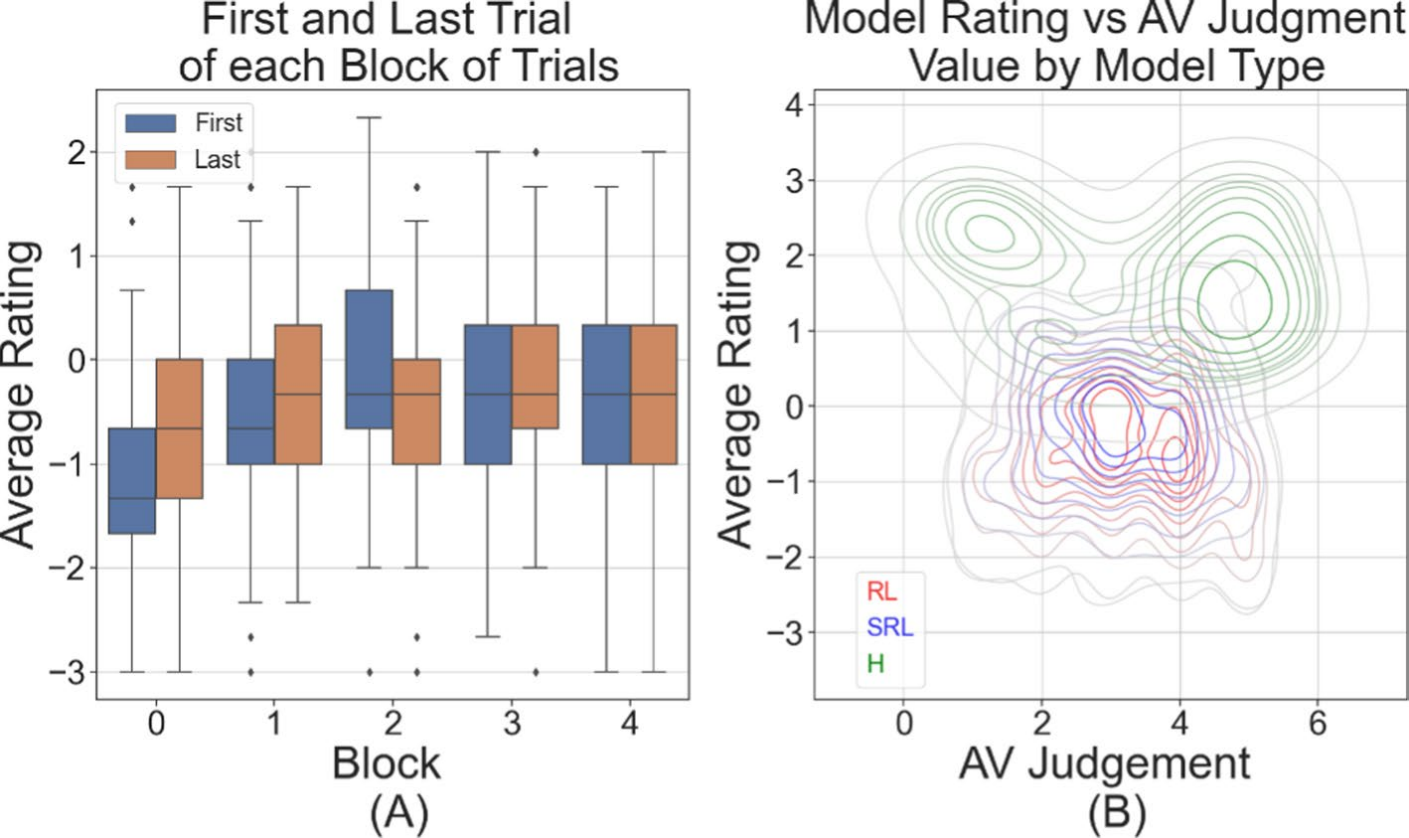
**a** Average over all 3 aspect ratings for the first and last trial in each block before the utility parameters were reset. There was an increase in rating of the resultant model at the end of the blocks, exhibiting large initial improvements but not in later blocks. **b** Contours of the averaged ratings of how likely the participants thought that instance was controlled by an AV, grouped by model type, with H being human-controlled, SRL being the fixed reward model, and RL being the utility reward model. Respective colours increase in intensity with higher contours relating to more observed instances in that region. H is clearly higher rated overall, but shows two distinct peaks over the AV judgment, indicating a divide of opinion amongst the feedback sample

What stands out prominently from the data is that most of the improvement, from the inception of the trials to their conclusion, was realized during the initial block. Figure 6a highlights this effect by showing the model rating at the first and last trial of each block, and that the final rating within a block significantly improved for the first 2 blocks but not for the final 3. Several factors could have caused these improvements to plateau. The model may have achieved the maximum improvement possible within the constraints of the given models. Alternatively, participants might be adjusting their expectations downward as they become more familiar with the system. Another consideration is the transient dynamics that emerge as the collective data settles into a state of equilibrium. Notably, the results indicate that the primary avenue for enhancing vehicle behaviour remains in the predictability of its movements. Erratic movement consistently emerged as a common feature among trajectories that were judged as lacking in safety aspects.

This diminishing return on improvement does, however, suggest that the model may have been approaching an optimal state, at least within the confines of the feedback provided, and even plausibly being rated poorly as it tried to move away from that state. The rapid initial improvement underscores the efficacy of the feedback mechanism in quickly refining the agent's behaviour, especially in the early stages of interaction. Such rapid initial refinement is advantageous and is the ultimate goal of this methodology. This ability to adjust its remit based on feedback is instrumental, putting the vehicle’s behaviour within the scope of human-accepted behaviour. This method is ideal to use before further refinement with more direct methods, such as relative preference ranking on shown trajectories, especially when considering that nuanced preferences might be pivotal for further honing the agent's behaviour.

For the fixed models presented during the 7th trial of each block, several observations were made. Firstly, the simple fixed model's performance remained consistent throughout the blocks, Fig. 6a. On an aggregate level, the fixed model ratings did not exhibit much variance $\left(Mdn=-0.333 , {\sigma }_{n-1}=0.9\right)$. The shaped model’s data distribution was still significantly different to the fixed model’s distribution, $Z(123)=-4.27,p=1.94\times {10}^{-5}$. This result suggests that, in terms of overall performance, there was not a discernible difference between the two models in the eyes of the participants, i.e., a poor shaped model instance and a fixed reward instance were not considered as different by participants.

A contrasting trend was observed when human-driven trajectories were introduced. Figure 6b displays the spread of judgments regarding the likelihood of a trajectory being controlled by an AV against their average rating across all 3 reward terms. The human trajectories were rated much higher overall and indicates multi-modal characteristics for the highly rated human-controlled trajectories. The policies learned by the RL agents were still very inferior to the human-driven ones, although a considerable amount of that effect is due to them being only partially trained policies. The human-driven trajectory’s multi-modality contrasts with findings from a previous experiment [20], where participants predominantly associated higher-rated judgements of trajectories with a greater likelihood of being autonomously driven. These trajectories consistently received superior ratings across all three evaluated aspects, in speed $$\left(Mdn=1 , {\sigma }_{n-1}=1.32\right)$$, position, $$\left(Mdn=2 , {\sigma }_{n-1}=0.789\right)$$, and steering $$\left(Mdn=2 , {\sigma }_{n-1}=1.37\right)$$, when compared to the AI-driven models ($$\sim H)$$, with an overall significantly increased median: $$Md{n}_{H}=1.67, Md{n}_{\sim H}=-0.67$$. The two-sided test between human-driven ($$H)$$ and Ai-driven models shows a significant difference $$Z(123)=-4.27, p=1.94\times {10}^{-5}$$ and remained significant when testing for the one-sided null hypothesis of $$H$$ having a greater rating, $$H>\sim H$$, $$Z(123)=20,p=1.41\times {10}^{-86}$$. These results suggest a perceptual bias or preference by people towards human-like behaviour or decision-making in the driving context. Yet, when participants were tasked with determining whether a particular trajectory was human-driven or AI-driven, the results were far from consistent. There is a notable variance in their judgments of these trajectories on average, as visible in the reported standard deviation values, covering the full spectrum of available ratings. This implies evidence for a new, nuanced interpretation: while a large cohort of participants did still equate superior performance with autonomous driving capabilities, there appears to be a distinct sub-group that held the opposite belief. Specifically, this latter group appeared to believe an exceptionally well-executed trajectory was more indicative of human control.

The variability of judgments regarding the likelihood of a trajectory being from an AV could be due to a multitude of influences, but further research in that area would be required. Yet, not all participants in this study shared the same viewpoint, especially for human-controlled trajectories as evidenced by their multi-modal characteristics. A distinct group appeared to lean towards the belief that human drivers would outperform AI in driving tasks. This split in perspectives further amplified the variability in judgments, underscoring the subjective nature of human perceptions when comparing AI and human driving behaviours. The repeated interactions, sub-group classifications, and aggregate statistics go part of the way to remedying the inherent fallibility of human feedback. Human judgements (and indeed expressed preferences) may not align with their true interests or quality criteria. The current method incorporates the subjective feedback in a direct numerical way and can optimise from feedback in an out-of-the-loop manner across multiple iterations. As a result, the method finds the reward shape that matches judgements/preferences the closest, not the judgements/preferences themselves, which allows for effects such as sample bias to propagate.

**Fig. 7:**
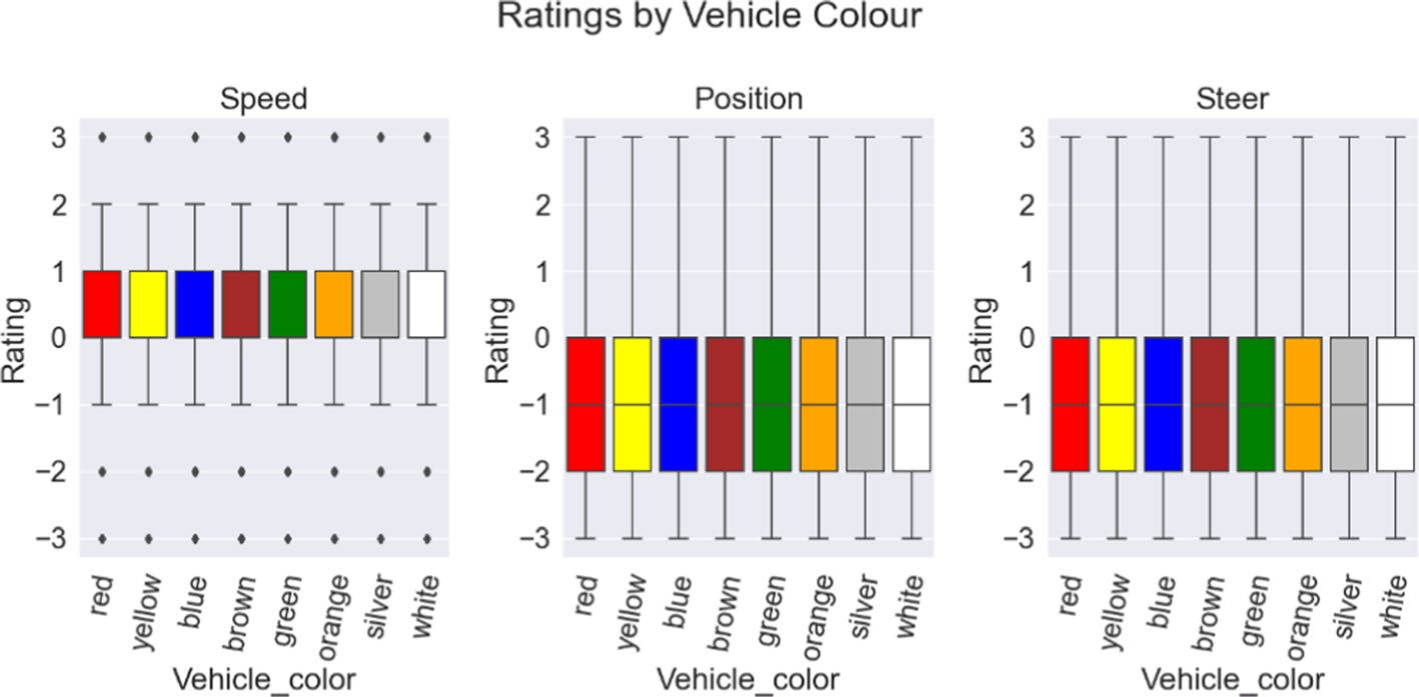
Ratings divided by colour and grouped by reward aspect. There were no observed differences based on the colour (Color figure online)

Regarding potential biases based on vehicle colour, the data did not reveal significant influence, all having $p=1$ for the null hypothesis of equality; the distribution of judgments remained uniform across all vehicle colours presented, Fig. 7. When the data was segmented into overly specific categories of colour ratings between model types and driving direction, some degree of variability does emerge; however, these variations were exclusively observed in categories that were poorly represented, having fewer than 500 trial instances. Given the limited sample size in these categories, the observed variability might be attributed to sample bias rather than any genuine effect related to vehicle colour.

Utility variants drove most of the vehicle trajectories observed, and a surprising 75% of these trajectories moved from left to right across the three seeds. It remains unclear why the ‘numpy.random.choice’ function, given only two directional options, exhibited this bias. Of the 50 trials, only 10 could deviate from this pattern, and for each of these 10 trials, there were four potential scenarios to select from: human-controlled trajectories moving left or right and fixed reward trajectories also moving left or right. By design, the policies were not fully converged. Despite this lack of convergence, a significant portion of the trajectories managed to approach their intended destinations closely. However, it was observed that many of these trajectories, although they were close to their goals, did not complete their tasks directly. Instead, they often meandered away from the destination, displaying a pronounced bias of moving upward and then returning to the centre. Interestingly, for the fixed model, the trajectories moving from right to left performed better. Yet, for the utility model moving right to left, they typically only managed to traverse about three-quarters of the intended path before their actions appeared to become random.

The ratings related to speed were predominantly centred around the mid-range of the scale. Speed ratings on the extreme were typically associated with trajectories that either did not head towards the goal (resulting in poor ratings) or those that deviated minimally from the path (resulting in favourable ratings), not clearly rating on speed alone. Steering ratings, on the other hand, were generally lower, and there was a distinct correlation whereby erratic trajectories, that is trajectories with frequent large changes in heading, received poorer ratings. The position term's ratings were in line with expectations; trajectories that strayed from an efficient path, especially those that deviated early on, were judged as having low safety. This demonstrates that some possible qualitative metrics of the trajectories are consistent with the broader findings regarding overall ratings. Regarding the pedestrian trajectories, their characteristics were uniformly distributed across all five rating bins and for all three rewards aspects. No discernible exploitative behaviour was detected in the pedestrian's movements. However, numerous trajectories displayed instances where the pedestrian seemed to halt or linger, suggesting that the participant might have been observing the vehicle's behaviour during these moments, rather than interacting naturally.

In the present work, not only are judgments elicited, but the participants are also fully in control of the pedestrian in the simulator using the keyboard. Allowing control of the pedestrian not only allows for an interactive experience and implicit understanding of the environment dynamics and vehicle behaviour, but also allows for the contrast of judgements reported with the behaviour of the participant. Such a comparison enables further analysis of the reported judgements; if a participant rates the vehicle as safe, do they act more confidently, or is there a contradiction between ratings and behaviour? No such trends were found in the data collected, but the interactive experiment allowed for their consideration.

Drawing insights similar to [21, 22], it becomes clear that accurately capturing the nuances of human preferences or judgments, especially as agents evolve towards more human-centric behaviours, is a formidable challenge. This complexity is evident in our results and collected data. For instance, while certain anticipated biases, such as those related to vehicle colour, were absent, unexpected biases emerged, such as the assessments regarding the likelihood of a trajectory being from an AV displaying a multi-modal distribution. These findings underscore the subtle nature of human perceptions and expectations. The existing literature corroborates that this domain offers a fertile ground for further investigation. The collected data still provides valuable insights, contributing to a deeper understanding of how AI models can be evaluated and improved through human feedback. The study paves the way for future research and development that can leverage human insights to create more effective and human-centred AI models, but also highlights the need for careful consideration in designing AI systems that not only perform well but also resonate with human intuition and values.

This approach delves deeper into the intricate dance between autonomous vehicles and human pedestrians, aiming to bridge the gap between computational advancements and human perceptions. By placing participants at the helm of the pedestrian's controls, we not only provide them with a more immersive experience but also capture a richer dataset, containing both explicit judgements and implicit behavioural nuances. The dual-layered approach offers a unique lens to scrutinise the alignment (or lack thereof) between a participant's actions and their stated perceptions. The ultimate goal is to craft autonomous driving policies that resonate with human expectations and instincts—intertwining the realms of behavioural science and reinforcement learning, with a view to ensuring that the future of autonomous driving is not just technologically advanced, but also intrinsically human-centric.

## Conclusion

This work attempts to shift the paradigm of policy evaluations to be more human-centric [22]. It leaves the efficient processing of observation data that accurately represents the vehicle’s environmental state to other work [6]. Instead, the work focuses on including the subjective human stakeholder’s opinions to improve automated driving performance in the social aspect of road use. This focus helps to address the simulation-to-real gap felt by subjective stakeholders in the real world, embedding automation into the social climate with minimal friction.

We have shown that incorporating the judgements of other road users can improve the performance in their subjective view. We do not push temporal credit assignment onto judgement makers, by only eliciting feedback on full trajectories of converged policies, instead transforming the already-shaped reward. The results show that an out-of-the-loop approach with iterative trials across many individuals helps give an overview picture of behaviours judged more favourably and allow for adaptive behaviours to better target a particular individual. For the latter, on-the-fly training may be possible with sufficient computation, which would alleviate the need for mass training of variants for the participant to effectively grid-search though the space of utility parameters.

Moreover, by leveraging repeated interactions, subgroup classifications, and aggregate data, we can address some of the inherent fallibility of human feedback. Participants' judgments (and derived preferences) might not always reflect their genuine interests or quality criteria. Our aim is not to claim a precise modelling of their choices. Instead, we have crafted a method that numerically integrates subjective feedback. By optimizing this feedback in an out-of-the-loop manner, we strive to align the reward shape with judgments/preferences as closely as possible, rather than replicating or modelling the generation of judgments/preferences themselves.

Future avenues could aim to delve deeper into the potential of generalizing and automating environmental reward shaping based on the judgments gathered [5] and preferences inferred. Testing the value of interacting with the AV and its effects on the subjects’ responses would also add to our understanding of the feedback interactions, possibly by running a comparative study with some no-interaction control groups [4, 14]. Furthermore, harnessing online policy optimization, i.e., ad hoc on-the-fly model training with adaptable feedback parameters, to iteratively refine judgment parameter estimates is thought to be enable further refinement. In summary, the in-depth questioning employed in the survey provided a holistic insight into how humans perceive AI-driven and human-driven behaviours in a simulated vehicular context and recognising behaviours that receive favourable judgments can pave the way for enhanced performance. Human demonstrations, preferences, or judgements are not only worthwhile to improve agent behaviour in complex environments, but also to assist in integrating automation into our lives.

## Footnotes

1 Code for the simulator is available at: https://github.com/Rik-Fox/pygame_ped_env.

2 All assets included with code at: https://github.com/Rik-Fox/pygame_ped_env/tree/master/pygame_ped_env/images.

3 Code available at: https://github.com/Rik-Fox/paavi.

4 Code adapted from: https://github.com/Stable-Baselines-Team/stable-baselines3-contrib and available at: https://github.com/Rik-Fox/pygame_ped_env/tree/master/pygame_ped_env/entities.

5 Code available at: https://github.com/Rik-Fox/experimentEagle22.
